# Examining the White Supremacist Practices of Funding Organizations for Public Health Research and Practice: A Composite Narrative From Female, BIPOC Junior Researchers in Public Health

**DOI:** 10.1177/15248399221129864

**Published:** 2022-10-29

**Authors:** Elizabeth Chen, Deshira Wallace, Cristina Leos, Yesenia Merino

**Affiliations:** 1 The University of North Carolina at Chapel Hill, Chapel Hill, NC, USA; 2 MyHealthEd, Inc., Chapel Hill, NC, USA

**Keywords:** Critical Race Theory, antiracism, White supremacy, grants, funding, professional development, early career researchers, junior researchers, BIPOC researchers, female researchers, health equity, health research, qualitative research, health promotion, career development, professional preparation

## Abstract

*Background*. It is challenging for junior public health investigators who identify as Black, Indigenous, or People of Color (BIPOC) to secure funding for projects and research. We used a narrative inquiry approach to understand and present the funding cascade from the perspectives of female, junior BIPOC researchers and provide funders with actionable recommendations to advance their antiracist goals. *Approach*. We applied a Critical Race Theory (CRT) framework to guide our narrative inquiry approach. The participants were the four co-authors and we each drafted individual narratives around our experience with the funding cascade and subsequently the five stages of narrative analysis. *Results*. We created a visual representation of key activities for funders and applicants organized by our perceived magnitude of inequities in a journey map, an interpreter table that describes common phrases and barriers encountered, and a composite counternarrative presented as a group text message conversation, elevating common themes including feeling pressured to have our research agendas conform to funders’ interests and receiving limited key information and support in the funding process. *Discussion*. We discussed how our findings represented manifestations of White supremacy characteristics like power hoarding and paternalism. *Implications for practice*. We offered specific antidotes for funding organizations to make their processes more antiracist and invited leaders of public health funding organizations to join us to further identify antidotes and share lessons learned in Fall 2023.

Public health funders often set research priorities—not researchers or practitioners. Members of these funding organizations, including board members, project officers, and grant reviewers, hold the power to decide which researchers, populations, issues, and methods receive emphasis and legitimacy. More recently, public health funders have increasingly expressed support for equity-focused projects (Denny, 2021; Fitbit, 2021; Robert Wood Johnson Foundation, 2021). These efforts, however, are limited by structural practices that maintain status quo including short turnaround times between announcing and closing requests for proposals and preferences to fund applicants who have received other competitive funding awards. Some of these structural practices may be the result of White fragility, epistemological ignorance, or racial bias among predominantly White decision-makers (Bowleg, 2021). Thought leaders have also critiqued how the discipline, its organizations, and public health professionals contribute to structural racism (Alang et al., 2021; Bowleg, 2021). Others outside of public health have challenged individuals to identify how White supremacy characteristics manifest in systems and to develop antidotes to them (Okun, 1999; Okun, 2021). We extend Okun’s work by critically examining funding practices in public health with the goal of igniting widespread change in the process.

It is challenging for junior public health investigators who identify as Black, Indigenous, or people of color (BIPOC) to secure funding for projects and research. A 2011 study of the National Institutes of Health (NIH) external research applications found that among Black and White applicants with comparably top-ranking scores, Black applicants were 13 percentage points less likely to be funded than White applicants (Ginther et al., 2011). A 2019 study found that some of this disparity may be due to research topics. Black scientists are less likely to be funded by the NIH than their White counterparts because they are more likely to develop research projects that focus on structural-level health determinants over individual-level outcomes (Hoppe et al., 2019). When examining the disparity in an intersectional way, women of color, specifically Black and Asian PhDs, and Black MDs, were significantly less likely be awarded an R01 compared with White women (D. K.Ginther et al., 2016). Latinx, Black, and Asian researchers are more likely to be new investigators (i.e., no previous R01 funding) than their White counterparts, thus less likely to be funded (Ginther et al., 2016).

Recent efforts by some organizations to improve equitable funding practices (Joseph et al., 2016) are necessary but insufficient to accomplish the structural changes required to level the playing field for individuals seeking funding for public health projects. We used a narrative inquiry to examine the experiences of female, junior (within 10 years of earning a doctorate degree) BIPOC researchers as they seek public health funding. Our goal was to illustrate how these experiences reflect manifestation of White supremacy characteristics and provide funders with actionable recommendations to work more directly toward their stated goals around equity in resources allocation.

Okun (2022) describes White supremacy culture among other things, as a constellation of historically and culturally situated characteristics embedded within mundane norms and standards of everyday life that “trains us all to internalize attitudes and behaviors that do not serve any of us.” Of particular relevance to this present analysis is Okun’s description of how these characteristics shape our ideas of professionalism (e.g., perfectionism, individualism, objectivity) and expertise within public health research and practice. In addition to presenting descriptions of these characteristics and how they show up in everyday life, Okun describes antidotes, or alternative ways of engaging with one another culturally as a means of refusing to comply with hegemonic norms that perpetuate White supremacy (e.g., understanding that our own world views and embedded assumptions shape our perceptions, thereby nullifying the notion that any person can be “objective”). We use Okun’s framework to organize our descriptions of how these White supremacist characteristics show up along the funding cascade (i.e., the series of applicant and funder processes related to applying for and awarding funding).

## Approach

We applied Critical Race Theory (CRT) as our framework (Crenshaw, 2011) to “center the margins” in our narrative inquiry approach. CRT states that racism is real, structural, and ordinary, meaning it permeates all facets of society (including the funding cascade) (Delgado & Stefancic, 2017). CRT provides a set of principles to bring race to the forefront, challenging notions of “race neutrality” or “color blindness.” CRT also calls for narratives and counter-storytelling to highlight the experiences of marginalized populations, paying particular attention to the diversity of experiences based on intersections between race, gender, class, sexuality, and other identities (Ladson-Billings, 2013). In addition, we drew from the work of Windsor et al. (2021) regarding the hidden curriculum, or unwritten rules and norms for academic advancement. Challenging the narrative of the “leaky pipeline” whereby BIPOC researchers are passively lost along the funding cascade, Liu et al. (2019) offer a counterview where academia creates and maintains “ladders” for those already functioning from a position of privilege to accelerate even faster up the ranks of academia and “chutes” wherein even the smallest misstep can slow or stop professional progress for those in more vulnerable positions (e.g., BIPOC researchers). We leverage this conceptualization in presenting our perspective of the funding cascade.

For this study, we used narrative inquiry to explore the lived experiences of female, BIPOC junior researchers pursuing US-based grant funding from federal and non-federal sources. Narrative inquiry focuses on each participant’s firsthand account and how they assign meaning to those events (S. R.Jones et al., 2013; Kim, 2020; Moen, 2016). We co-constructed a composite counternarrative in which we highlight how we navigate the funding process (Solórzano & Yosso, 2016). Composite counternarratives present data that reflect the experiences of those who hold shared social identities and experiences within a particular context (Griffin et al., 2014). While these experiences are not to be generalized to all persons who share these identities, these experiences can highlight common experiences across a larger marginalized group.

### Participants

Participants of this narrative inquiry are the co-authors—all junior public health researchers who identify as BIPOC women, some of whom are also first-generation scholars. We leveraged the gendered, racialized, and professional statuses we experience to interrogate how systematic differences occur along the funding cascade.

### Analysis

**Figure 1:**
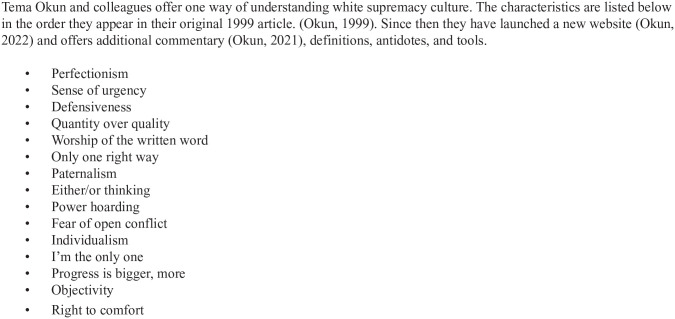
Characteristics of White Supremacy Culture

As a group we began by discussing our individualized experiences with obtaining federal and non-federal research funding, then two co-authors created a timeline based on these experiences (Figure 1). This basic timeline—or *journey map*—was constructed iteratively as a group as we built a collective understanding of the journey toward obtaining research funding. Using the initial iteration of the journey map, we developed five questions for each co-author to answer independently in written narratives. Sample questions included “What unspoken rules have you learned along the way?” and “How did your intersectional identities affect your experiences seeking research funding?”

We then followed the five stages of narrative analysis (Crabtree, 1999) to move from individual narratives to a composite counternarrative. First, each author read another co-author’s narrative and noted their impressions via memos. Second, we met in person to discuss impressions across narratives. Third, we read the other individual narratives to identify themes, additional decision points, barriers, facilitators, and points for intervention. Fourth, we collectively drafted four analytic products that were used iteratively to develop the composite counternarrative. We developed a composite narrative to highlight a snapshot of what conversations may look like when seeking funding, a journey map to present a larger view of the funding cascade, an interpreter table presenting what reviewers can say and how they are interpreted (Table 1), a barriers and facilitators matrix (Table 2), and the chutes and ladders schema (Supplemental Appendix A). These products were co-created across multiple rounds of review and were informed by the other. The journey map, barriers and facilitators matrix, and composite narrative are presented below.

**Table 1:** Interpreter Table: Decoding Funder Comments for Hidden Meanings and Relevant Antidotes to White Supremacy Characteristics

What is said by funders . . .	What we thought it meant . . .	What it actually means . . .	Antidotes of manifestations of White supremacy characteristics . . .
What’s the health outcome?	• Health outcome can be defined broadly and include outcomes at multiple socio-ecological levels	• Health outcome needs to be drilled down to the individual/biological level	**Either/Or Thinking** • Refrain from over-simplifying; call for proposals that account for complex ideas to address complex problems
Idea is not the right “fit”	• Revamp the proposal to showcase upstream, public health solutions to address cross-cutting issues • Keep searching for other funding bodies that might be a “better fit”	• Proposals that address cross-cutting structural determinants have a hard time finding a funding home, unless the proposal is simplified to address a down-stream issue • Not considered traditional public health proposals • Funders have narrowly defined priorities that are not shared publicly	**Paternalism** • Specify desired outcomes without pre-specifying solutions so applicants can be creative • Create more open-ended opportunities that allow for more bottom-up/community-driven activities
Research is unfundable	• Support is available to receive feedback on ways to move ideas toward a more “fundable” proposal that is still true to the idea	• Given little to no feedback on ways to adjust proposal • Give up on the work that drives you to pursue more “fundable” options	**Defensiveness** • Share scoring criteria/rubrics with applicants • Provide specific feedback to applicants to competitiveness for future funding
Be innovative	• Think outside the box • Engage in the literature that may be underdeveloped or missing critical perspectives	• Be incrementally innovative • Need to show sufficient evidence, even if the evidence is neither relevant nor of high quality, particularly when proposing approaches to improving health equity • “Controversial ideas” (these vary based on time and funders) such as those focused on structural determinants may not be funded	**Quantity Over Quality** • Be open to explicitly fund projects that justified different kinds of data besides datasets or previously published work
Too much jargon	• Edit the content to be clearer for a non-expert to understand and ensure that the rationale is logical	• Terms or ideas that critically push forward ideas and methodologies can engender asks for “tampering down” the language in the grant proposal • Unwillingness on behalf of funders to critically examine their (mis)understanding of terms and research areas, particularly anti-racist and social justice–related work • “Objective” reason to justify rejection of ideas that make funders uncomfortable	**Right to Comfort** • Learn to sit with discomfort before reacting • Deepen analysis of racism and oppression to increase personal and professional understandings to more equitably review proposals
Be a “competitive applicant”	• Publish an unspecified amount of peer-reviewed publications • Showcase grant-writing experience (i.e., internal grants)	• Have secured external grants before applying • High levels of “productivity” in the form of peer-reviewed publications • Have previous experience doing the exact thing you are proposing to do	**Perfectionism/Qualified** • Focus on the quality of the applicant to do the proposed work rather than unspecified and often bias “failures” that are tied to inaccurate notions of objectivity
Build a diverse team	• Construct diverse teams according to lived experiences, identities, and education levels which can add layered and diverse expertise	• Select collaborators with certain degrees and titles to delineate “formal” expertise, thus increasing likelihood of being awarded	**One Right Way/Worship of the Written Word** • Promote shared leadership among “traditional” PIs (e.g., PhDs) and non-traditional PIs for grant submissions
Open RFPs	• Broad ideas are welcome for review • Focus of application, application requirements, and timeline for submission is clear	• Hidden priorities may reduce eligibility of potential candidates • RFPs may be specific to the work of specific organizations and not actually “open” for all candidates	**Power Hoarding** • Reduce barriers to information sharing, including transparency regarding the key priorities targeted for the RFP
Closed RFPs	• Reaching out to the funders to build a relationship to determine potential present/future opportunities	• Pathways for learning of opportunities are obscure • Candidates are invited based on existing relationships; however, relationships are often built on who you know thus remain homogeneous (often White)	**Power Hoarding** • Invest intentionally in building relationships with HBCUs, HSIs, Tribal Colleges, and community organizations. This includes personalized outreach and investment into creating or strengthening research infrastructure, among others
Network/establish relationships with the funders	• Calling, emailing, meeting with points of contact early and often • There is a reciprocity in relationship building between funders and candidates	• Responsiveness of program officers or points of contact vary • Onus is on candidate, particularly those of “diverse” backgrounds to foster the relationship(s) rather than for the funders to facilitate opportunities for relationship-building	**Power Hoarding** • Facilitate equitable, consistent access to program officers • Clearly describe organization communication style and expectations

*Note.* PI= principal investigator, RFP = request for proposal; HBCU = Historically Black College and University; HSI = Hispanic-serving institution.

**Table 2:** Barriers, Facilitators, and Places to Intervene Along the Funding Cascade

Facilitators or barriers	Opportunities for intervention
Facilitators	
Self-reliance • Individual self-reliance and problem-solving skills have allowed for successes despite not having full access to knowledge and resources regarding the grant process. • Note that self-reliance and problem solving is a response to consistently being in environments where information is withheld or there are assumptions of what is “common knowledge” or not.	1. Self-reliance is a valuable skill for all researchers/practitioners; however, institutions/centers/foundations can leverage their current information channels and improve the readability and accessibility of this information for all.
Peer knowledge and support • Peers are often sources of information and expertise, and provide spaces for rich discussions of ideas and problem solving when other sources of “support” or “information” fail	1. Funders can facilitate spaces for potential grantees to build a peer network and share information. a. These spaces can be online. Think of a more interactive NCFDD (National Center for Faculty Development and Diversity) 12-week writing group. Ideally, these spaces would be subsidized at low or no cost to early career participants to not perpetuate disparities in access to information/resources. 2. Institutions or for smaller organizations, a developed consortium could facilitate peer-to-peer data sharing (can be grant funders).
Collaboration with aligned funders • Once funders or grantors that are receptive to the types of research or approaches are found, a more focused approach can be taken to target these specific opportunities. However, there may still be ambiguity with fit.	1. Funders can collaborate with other funding agencies to identify individuals or organizations working in their priority area, particularly those that have not previously received funding.
Clear instructions and flexible processes • Simple, clear application process that acknowledges ideas can and sometimes should change based on new learnings and contextual circumstances. • Ability to pivot from conceptualization to implementation of ideas (e.g., Innovation Next) that reduced bureaucratic hurdles	1. Funders can provide a simple application process to capture the most important aspects of a proposal idea, rather than focusing on extremely detailed proposals that are created without a true understanding of the future state in which this work will be done. 2. Allow researchers to pivot their work plans easily without excessive justification or administrative burden to explain why they are deviating from the original proposal plan.
Barriers	
Unwritten rules The unwritten rules of grant writing where: • The rules are unclear • The rules keep changing • The grant landscape changes often and without warning • The gatekeepers, or holders of key information, control who gets access to that information and when These unwritten rules require high levels of effort to gear up for each opportunity with little reward	1. Funders improving transparency on rules and procedures would reduce the amount of labor needed to find necessary information.
Vague information mechanisms • Not having clear pathways to receiving information and learning the norms of grantsmanship from advisors, mentors, and funders meant there were significant inefficiencies (wasted time) especially for first-generation graduate students and researchers	1. Current outreach on grant opportunities is challenged because of assumptions that modes of information sharing are the same for all early-career researchers, rather than accounting for smaller or more fractured professional networks for researchers that experience multiple oppressed identities in addition to their early career status. Active outreach is needed that includes building broader and deeper networks outside of traditional spaces (e.g., R1 institutions, large non-profits) to increase information sharing for opportunities and technical support. 2. Funders hosting intentional onboarding activities to address funding processes so that early-career, first-generation, and ethnoracially minoritized researchers gain big picture understanding and start to understand what questions to ask of their institutions. Examples include application instructions, portals, and contact information; who needs to be involved in preparing and submitting proposals; resources to support administrative tasks; and understanding overhead.
Networks of exclusion • The norms of grantsmanship may look particularly different for foundation-type grants, where learning of opportunities may come directly from an undefined yet exclusionary network, the entryway into these foundations is more obscure than for some large foundations or large government funding mechanisms • Segregation of philanthropic social networks leads to inequitable access to foundation funding for Black, Indigenous, Latinx, Asian and Asian American, women, non-binary individuals, and LGBTQIA+ communities. Yet much of the funding around health disparities or health inequities affects these very same populations.	1. Grantors have the power to create; these improve access to these communities beyond one-time grant opportunities to “improve diversity.” 2. Foundations and non-governmental sources of funding may be open or closed RFPs. Funders that use closed RFPs, which require invitations to apply, need to be more proactive in the expansion of their network of potential grantees.
The “scope” trap • Limitations on the “scope” or operationalization of public health research as health outcome focused, and not by cross cutting structural issues that affect well-being • Ideas that improve the well-being of people, but do not fit narrowly defined missions are difficult to find “funding homes” in, therefore, more senior researchers or funders may discourage the cross-cutting work that is needed. This limitation in funding sources and types at the early part of the careers of WOC can initiate the segmentation of researchers into “fundable” and “non-fundable” rather than focusing on innovation and impact.	1. Reallocate funding that addresses structural issues (i.e., racism, wealth distribution) and are reflective of the complexities of human experiences (i.e., individuals do not only experience one thing at a time). a. NIMHD (National Institute on Minority Health and Health Disparities) is one of the least funded I/C at NIH (National Institutes of Health); however, in increasing their funding (doubling or more) there would be greater opportunities to develop studies that address cross-cutting issues rather than be focused on one health outcome or behavior. b. Increase the number of RFPs that are collaborative across current I/Cs as a place of synergy to address social/structural determinants in a more comprehensive way.
The need for data • Proposing research ideas that require pushing against the status quo without formative research/data is a challenge. Grant reviewing convention is that data are needed to prove a point; however, if the data do not exist to address inequities, and funds will not be released until there are data, this causes an endless loop segmenting out innovation for the sake of perceived fundability.	1. Develop new grants or restructure grant criteria that evaluate innovation and impact.
The limited time for RFPs • Quick turnarounds between the announcement and submission date systematically excludes potential applicants who do not already have the systems in place to respond quickly.	1. Grantors have the power to improve transparency around grant due dates. This includes: a. The number of grants they plan to fund. b. If the grant opportunity will be recurring (e.g., 1 time opportunity or available for X number of years) to allow for better strategic planning. 2. Establish a minimum length of time between when funding opportunities are announced and the deadlines
Extended timing for reviews and awards • On the other side, the (sometimes) long wait between submission, review, and distribution of funds may not coincide with the needs of the stakeholders, particularly if the grant is community focused.	1. Develop processes that allow for timely review of proposals once they have been submitted.
The review process • The ideal is to receive constructive feedback that can improve the proposed grant idea, which can be especially helpful for early career/junior researchers and practitioners of color. The reality is that some comments can be constructive, but others are not specific, incorrect indicating that the reviewer did not read the grant in any detail, or just a matter of opinion. Comments like these do not allow much room for improvement. Yes, there can be edits to clarify language, but when subjective comments arise about the qualifications of the applicant there is no amount of editing that will change that. • Foundation grant reviews often do not provide any comments at all, which makes it difficult to understand the areas to improve the grant proposal in the future	1. Funders can make a commitment to providing constructive feedback for proposals they receive. This applies even if it means developing a standardized way of giving feedback at scale. 2. Allocate the proper resources necessary to conduct a proper review of proposals, including funding, training, and sufficient time. 3. Those tasked with reviewing and evaluating proposals must have content area expertise that is relevant and not just tangential, in addition to displaying competencies that support the delivery of constructive feedback (i.e., coachability, clear communication)

*Note.* RFP = request for proposal, WOC = women of color.

## Results

**Figure 2:**
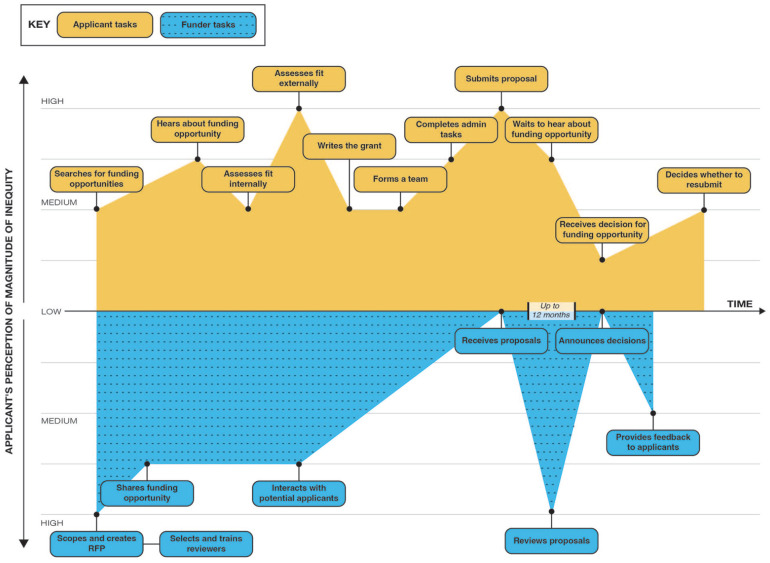
Funding Journey Map by Role, Time, and Perceived Magnitude of Inequity *Note.* RFP = request for proposal.

The funding journey map (Figure 2) provides a visual representation of key activities for funders and applicants along the funding cascade. From the applicants’ perspective, the journey map begins with searching for funding opportunities and includes moments in-between like writing the grant, submitting the proposal, waiting to hear, and receiving a decision, and then ending with deciding whether to resubmit a proposal if denied. From the funders’ perspective the journey map begins with creating request for proposals (RFPs) and includes moments in-between like interacting with potential applicants, receiving proposals, reviewing proposals, announcing decisions, and then ending with providing feedback (or not) to applicants.

The journey map depicts our collective perceived magnitude of inequities for each activity as low, medium, or high. “Low” activities show little to no patterns of structured differential access and has minimal impact on funding outcomes, “medium” activities show some patterns of structured differential access and has a moderate impact on funding outcomes, and “high” activities show clear differential access by identity groups and have a large impact on funding outcomes. Most activities were deemed to have medium or high levels of inequities, with only three activities identified as having low inequities: receiving a funding decision (for applicants) and receiving applications and announcing funding decision (for funders).

The interpreter table (Table 1) describes common phrases encountered when preparing and submitting funding proposals. These 10 phrases reflect common reasons for denying funding, coded as “objective” feedback. In reality, these phrases have alternate meanings imbued with characteristics of White supremacy. For example, the notion of “fit” is often used to obscure reviewer or funder biases. A subjective “fit” could be a matter of a research topic being investigated in a way in which a reviewer or funder is familiar, and therefore comfortable, with rather than any actual misalignment with the stated purpose of a funding mechanism. Similarly, an innovative approach to a public health issue may be seen as an ill “fit” because it centers alternative knowledges (e.g., BIPOC scholars with less notoriety than their White counterparts, community-grounded forms of expertise) rather than those with which reviewers or funders are more familiar. However, in receiving vague feedback about a specific research or project idea not being the right “fit,” a junior BIPOC scholar is left with an unclear understanding of how to either target funding mechanisms that are more open to this form of inquiry or how to reconceptualize a project to fit normative standards. This table serves to explicitly describe some of the practices that implicitly maintain inequitable funding outcomes.

Table 2 describes additional facilitators and barriers encountered at various parts of the funding cascade. We identified four facilitators that could be formally incorporated into institutions to support more equitable funding outcomes for female, BIPOC junior researchers: (1) self-reliance, (2) peer knowledge and support, (3) collaboration with aligned funders, and (4) clear instructions and flexible processes. We also identified eight barriers within the funding cascade that represent key areas for intervention: (1) unwritten rules, (2) vague information mechanisms, (3) networks of exclusion, (4) the “scope” trap, (5) the need for data, (6) the limited time for request for proposals, (7) extended timing for reviews and awards, and (8) the review processes. The chutes and ladder figure (Supplemental Appendix A) summarizes key moments when BIPOC junior researchers may exit academia.

**Figure 3:**
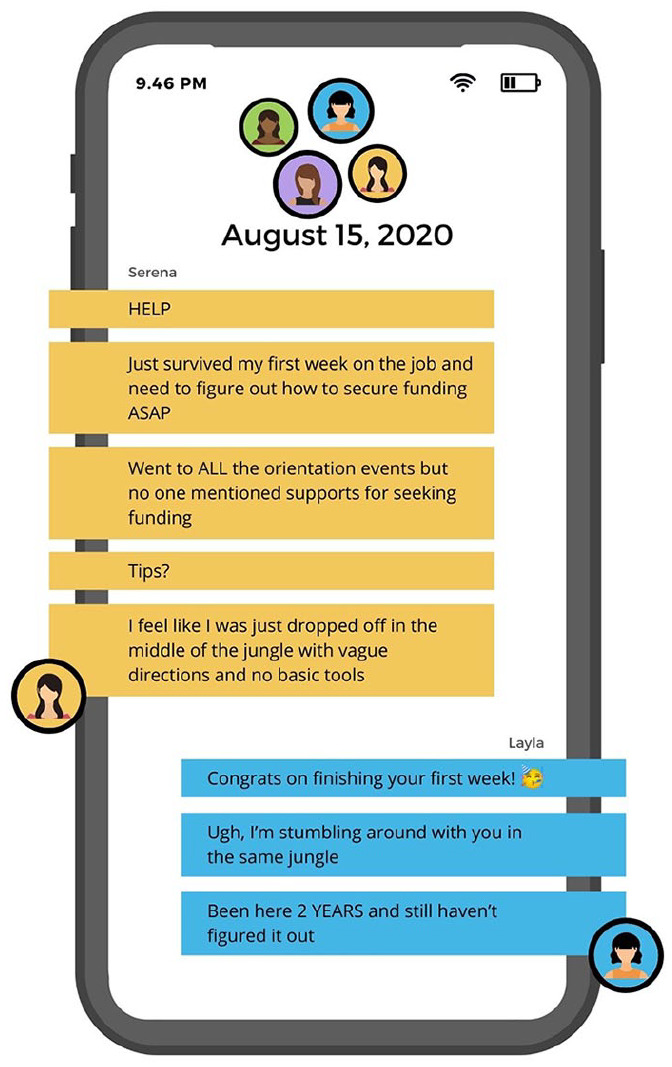
Composite Narrative Via a Group Test Message Thread Part 1

**Figure 4:**
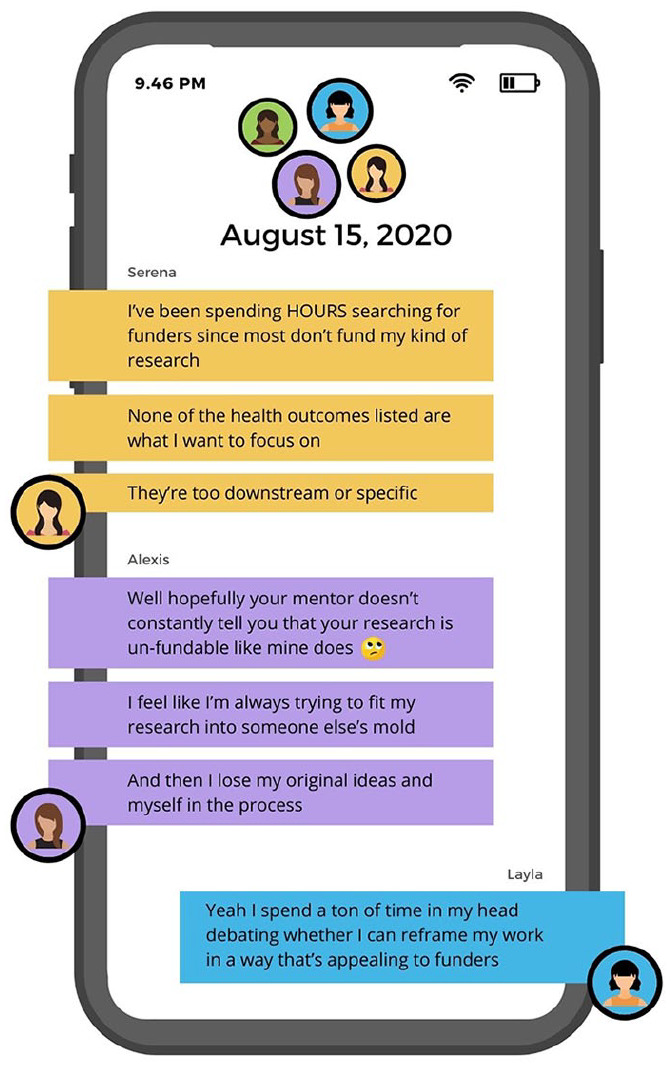
Composite Narrative Via a Group Test Message Thread Part 2

**Figure 5:**
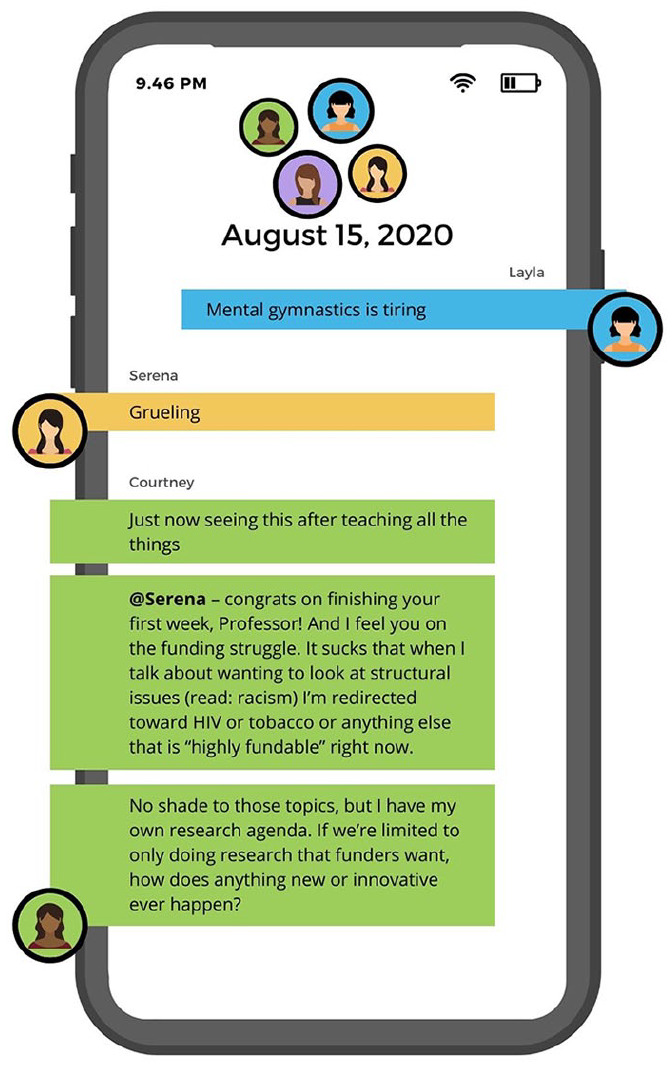
Composite Narrative Via a Group Test Message Thread Part 3

Our composite counternarrative is presented as a fictional group text message thread to represent how characters sought funding advice and information through informal channels. The discussion is initiated by Serena, a new faculty member at a large research (R1) university. She was recently hired as a fixed-term assistant professor for which she is expected to cover 70% to 90% of her salary through grants and contracts. The friends depicted are female, BIPOC junior researchers, first-generation academics, and graduated with doctorates within the last 3 years. The group discusses their respective challenges seeking and securing funding (Figures 3–5). See the full text chain in the Supplemental Materials.

## Discussion

### Shared Experiences

While the authors of this paper have varied research interests and career goals, there are many experiences that we shared as female, BIPOC junior researchers that we captured in the journey map, interpreter table, and composite counternarrative above. Some of these shared experiences and feelings include the following:

- Feeling our research ideas are undervalued;
- Hearing that our research, while innovative, does not align with strategic priorities of funding organizations;
- Feeling pressured to conform our research agendas to funders’ interests;
- Receiving limited key information during or after the funding process;
- Watching White and more affluent colleagues navigate academic and funding structures more easily;
- Feeling a large disconnect between community needs and funder requirements.

The funding cascade is made up of multiple systems at play that lead to—and reproduce—systematic differences with regard to funding outcomes.

### Manifestations of White Supremacy Culture and Implications for Practice

Based on our narrative inquiry, we expand on some of the more salient components of White supremacy culture that emerged, grouped in what we saw as clustering of mutually reinforcing characteristics. This list is not intended to be exhaustive of the manifestations of White supremacy culture that may appear throughout the funding cascade.

#### Power Hoarding and Paternalism

The mechanisms for hoarding power and resources are vast along the path to obtaining grant funding. Grants, especially federal and national, non-federal ones, tend to be awarded to those who have already successfully competed for grants. Even grants geared toward junior researchers are typically framed under the mentorship of those who have successfully joined that circular stream of previously funded leading to subsequent funding. This mechanism of allocating funding and power to the same people encourages the multigenerational reproduction of the same views on persistent health issues. This does little to foster innovation, which ironically many RFPs claim to encourage.

Related to power hoarding, we saw that many facets of the funding process showed classic operationalizations of paternalism. Using Okun’s definition of paternalism as a facet of White supremacy culture (Okun, 1999), those in positions of power know the specific rules of decision-making and do not see it necessary to convey those rules to those impacted by their decisions. This is seen in the vague instructions on RFPs, the limited feedback provided to grant applicants, and the obscurity with which funding priorities are set, relationships with funders are established, and the dearth of entry points into funding streams for BIPOC junior researchers.

Okun’s (2021) antidotes to power hoarding and paternalism can be operationalized along the funding cascade by:

- Moving away from a narrow view of how health research should be conducted to foster greater innovation;
- Expanding representation of who can serve on review boards for open grants to foster broader perspectives;
- Increasing access to information for those who are not in positions of power within decision-making processes.

Power hoarding and paternalism are experienced every day in the form of a lack of transparency of and noting how funders dictate what is worthy of funding, for how much funding, and the length of funding. For instance, having one-time funding opportunities to improve structural health inequities will not address the long-standing exclusionary practices that caused the disparity in the first place. Rather funders need to shift power in who is developing the calls for funding to those who have the context area expertise health disparities and/or lived experiences of navigating structural inequities, be more proactive in the expansion of networks for potential grantees, and improve transparency across the grants process.

#### Sense of Urgency, Perfectionism, and Quantity Over Quality

There are few places where the sense of urgency has been easier to observe than recent attempts to introduce health and racial equity into funding portfolios. These RFPs have on average even shorter turnaround times than other funding opportunities, which do not account for the structural disadvantage experienced primarily by women who are often primary caretakers of their families, offer fewer funding dollars, and require unrealistic time frames for creating structural change that diminish the possibility of meaningful engagement with communities most affected by health inequities and racism. Moreover, folding restrictive windows of opportunity from funding announcement to submission deadline into calls for equity and inclusion is antithetical to the very antiracist programs they seek to fund. Antiracist approaches require additional effort, and therefore additional time, to engender inclusive grant development practices that foster trust and collaboration necessary to achieve antiracist goals.

Paradoxically, this sense of urgency puts White supremacy tenets of perfectionism and quantity over quality at odds with one another. While there is an emphasis in grant writing on having as close to perfect a grant proposal as possible for the most competitive application, the need to apply to many grants quickly makes meeting those high standards for quality exceedingly difficult to attain. As junior researchers, we have all gotten the messaging that the only way to win grants is to continually apply for as many as you can. Recent shifts at some institutions, however, have shown that an increased focus on quality grant development, including structural support for junior faculty, increased researchers’—especially BIPOC researchers—success in their scholarship endeavors (McBride et al., 2019).

Okun’s (2021) antidotes to sense of urgency, perfectionism, and quantity over quality can be operationalized along the funding cascade by:

- Allowing more time from announcing an opportunity for grant funding to when application deadlines are due, particularly for those that seek to privilege community-engaged methods and knowledge;
- Including consideration of how research has engaged with, will communicate with, and impacts communities most affected by the given topic under study;
- Developing guidelines for how funded projects will name, learn from, and leverage inevitable mistakes as they conduct research;
- Prioritizing proposals that keep sustainability in mind, namely research that impacts health seven generations into the future.

The implications of these characteristics of White supremacy include that innovative ideas go unsubmitted and thereby unfunded. In addition, BIPOC junior researchers who are carving out new ideas can experience barriers such as the working with a limited scope of what are considered fundable public health topics (i.e., scope trap), the need to show preliminary data when those data likely do not exist for projects centered on health equity, or trying to navigate exclusionary and segregated systems of information particularly for foundation grants, opportunities can be learned from undefined and exclusionary networks. If we are not aware of and a part of these networks, there will always be inequitable access to funding, even if the funding opportunities are focused on the communities we are a part of and work with. As grants funding is an increasingly critical component for job security and advancement in multiple sectors, substantial change is needed to address the structural barriers inherently embedded in the process. Without informed changes the chutes will continue to grow, making it harder for BIPOC junior researchers to stay in their research organizations and contribute their innovative ideas that push the status quo toward solutions that improve the well-being of communities affected by racism and other structural inequities.

We have used the tenets of White supremacy culture in discussing the funding cascade as an aperture to shine light on ways in which the existing systems work to disadvantage female, BIPOC junior researchers specifically and all knowledge creation efforts more broadly. In doing so, we hope to catalyze engaged parties in conceptualizing, designing, and implementing the structural changes necessary to move the field of health research toward actively antiracist funding modes of operation.

### Call to Action

In an effort to transform the funding cascade, we invite public health funders to join us for a series of virtual convenings in Fall 2023 hosted by the UNC Gillings School of Global Public Health so that funders can collectively:

1. Identify problematic practices using Okun’s (2021) updated White supremacy characteristics as a starting point, and integrating existing data, including narratives, of the experiences of BIPOC junior researchers at their institutions and the institutions they fund;
2. Identify potential solutions and antidotes;
3. Forecast resources needed to enact these changes and potential barriers to change;
4. Crowdsource technical assistance or support.

To receive updates about these convenings, to invite a funder to the convening, or to submit a comment to the co-authors, please fill out this form: https://bit.ly/antiracistfunding. In the meantime, we ask that funders internally reflect on action items 1 through 4 above and hold themselves accountable to making the funding cascade more antiracist. While the focus of these results and recommendations is funders, there are also many instances where other institutions (e.g., academic institutions, academic journals, professional networks) limit the potential of BIPOC researchers. We encourage members of these other systems reflect on individual and collective practices that must be transformed to drive toward equity.

## Conclusion

We share the perspectives of female, junior BIPOC researchers navigating the grants landscape. The funding cascade is filled with barriers that maintain the status quo and are reflective of continued normalization of White supremacy culture. Structural barriers to the grants process require structural solutions as a means of improving equity in the workforce, equity in research being conducted, and improved equity in public health.

## Supplemental Material

sj-jpg-1-hpp-10.1177_15248399221129864 – Supplemental material for Examining the White Supremacist Practices of Funding Organizations for Public Health Research and Practice: A Composite Narrative From Female, BIPOC Junior Researchers in Public Health Click here for additional data file. Supplemental material, sj-jpg-1-hpp-10.1177_15248399221129864 for Examining the White Supremacist Practices of Funding Organizations for Public Health Research and Practice: A Composite Narrative From Female, BIPOC Junior Researchers in Public Health by Elizabeth Chen, Deshira Wallace, Cristina Leos and Yesenia Merino in Health Promotion Practice

sj-jpg-10-hpp-10.1177_15248399221129864 – Supplemental material for Examining the White Supremacist Practices of Funding Organizations for Public Health Research and Practice: A Composite Narrative From Female, BIPOC Junior Researchers in Public Health Click here for additional data file. Supplemental material, sj-jpg-10-hpp-10.1177_15248399221129864 for Examining the White Supremacist Practices of Funding Organizations for Public Health Research and Practice: A Composite Narrative From Female, BIPOC Junior Researchers in Public Health by Elizabeth Chen, Deshira Wallace, Cristina Leos and Yesenia Merino in Health Promotion Practice

sj-jpg-11-hpp-10.1177_15248399221129864 – Supplemental material for Examining the White Supremacist Practices of Funding Organizations for Public Health Research and Practice: A Composite Narrative From Female, BIPOC Junior Researchers in Public Health Click here for additional data file. Supplemental material, sj-jpg-11-hpp-10.1177_15248399221129864 for Examining the White Supremacist Practices of Funding Organizations for Public Health Research and Practice: A Composite Narrative From Female, BIPOC Junior Researchers in Public Health by Elizabeth Chen, Deshira Wallace, Cristina Leos and Yesenia Merino in Health Promotion Practice

sj-jpg-2-hpp-10.1177_15248399221129864 – Supplemental material for Examining the White Supremacist Practices of Funding Organizations for Public Health Research and Practice: A Composite Narrative From Female, BIPOC Junior Researchers in Public Health Click here for additional data file. Supplemental material, sj-jpg-2-hpp-10.1177_15248399221129864 for Examining the White Supremacist Practices of Funding Organizations for Public Health Research and Practice: A Composite Narrative From Female, BIPOC Junior Researchers in Public Health by Elizabeth Chen, Deshira Wallace, Cristina Leos and Yesenia Merino in Health Promotion Practice

sj-jpg-3-hpp-10.1177_15248399221129864 – Supplemental material for Examining the White Supremacist Practices of Funding Organizations for Public Health Research and Practice: A Composite Narrative From Female, BIPOC Junior Researchers in Public Health Click here for additional data file. Supplemental material, sj-jpg-3-hpp-10.1177_15248399221129864 for Examining the White Supremacist Practices of Funding Organizations for Public Health Research and Practice: A Composite Narrative From Female, BIPOC Junior Researchers in Public Health by Elizabeth Chen, Deshira Wallace, Cristina Leos and Yesenia Merino in Health Promotion Practice

sj-jpg-4-hpp-10.1177_15248399221129864 – Supplemental material for Examining the White Supremacist Practices of Funding Organizations for Public Health Research and Practice: A Composite Narrative From Female, BIPOC Junior Researchers in Public Health Click here for additional data file. Supplemental material, sj-jpg-4-hpp-10.1177_15248399221129864 for Examining the White Supremacist Practices of Funding Organizations for Public Health Research and Practice: A Composite Narrative From Female, BIPOC Junior Researchers in Public Health by Elizabeth Chen, Deshira Wallace, Cristina Leos and Yesenia Merino in Health Promotion Practice

sj-jpg-5-hpp-10.1177_15248399221129864 – Supplemental material for Examining the White Supremacist Practices of Funding Organizations for Public Health Research and Practice: A Composite Narrative From Female, BIPOC Junior Researchers in Public Health Click here for additional data file. Supplemental material, sj-jpg-5-hpp-10.1177_15248399221129864 for Examining the White Supremacist Practices of Funding Organizations for Public Health Research and Practice: A Composite Narrative From Female, BIPOC Junior Researchers in Public Health by Elizabeth Chen, Deshira Wallace, Cristina Leos and Yesenia Merino in Health Promotion Practice

sj-jpg-6-hpp-10.1177_15248399221129864 – Supplemental material for Examining the White Supremacist Practices of Funding Organizations for Public Health Research and Practice: A Composite Narrative From Female, BIPOC Junior Researchers in Public Health Click here for additional data file. Supplemental material, sj-jpg-6-hpp-10.1177_15248399221129864 for Examining the White Supremacist Practices of Funding Organizations for Public Health Research and Practice: A Composite Narrative From Female, BIPOC Junior Researchers in Public Health by Elizabeth Chen, Deshira Wallace, Cristina Leos and Yesenia Merino in Health Promotion Practice

sj-jpg-7-hpp-10.1177_15248399221129864 – Supplemental material for Examining the White Supremacist Practices of Funding Organizations for Public Health Research and Practice: A Composite Narrative From Female, BIPOC Junior Researchers in Public Health Click here for additional data file. Supplemental material, sj-jpg-7-hpp-10.1177_15248399221129864 for Examining the White Supremacist Practices of Funding Organizations for Public Health Research and Practice: A Composite Narrative From Female, BIPOC Junior Researchers in Public Health by Elizabeth Chen, Deshira Wallace, Cristina Leos and Yesenia Merino in Health Promotion Practice

sj-jpg-8-hpp-10.1177_15248399221129864 – Supplemental material for Examining the White Supremacist Practices of Funding Organizations for Public Health Research and Practice: A Composite Narrative From Female, BIPOC Junior Researchers in Public Health Click here for additional data file. Supplemental material, sj-jpg-8-hpp-10.1177_15248399221129864 for Examining the White Supremacist Practices of Funding Organizations for Public Health Research and Practice: A Composite Narrative From Female, BIPOC Junior Researchers in Public Health by Elizabeth Chen, Deshira Wallace, Cristina Leos and Yesenia Merino in Health Promotion Practice

sj-jpg-9-hpp-10.1177_15248399221129864 – Supplemental material for Examining the White Supremacist Practices of Funding Organizations for Public Health Research and Practice: A Composite Narrative From Female, BIPOC Junior Researchers in Public Health Click here for additional data file. Supplemental material, sj-jpg-9-hpp-10.1177_15248399221129864 for Examining the White Supremacist Practices of Funding Organizations for Public Health Research and Practice: A Composite Narrative From Female, BIPOC Junior Researchers in Public Health by Elizabeth Chen, Deshira Wallace, Cristina Leos and Yesenia Merino in Health Promotion Practice
