# Supplementary figures and images for: Examining the White Supremacist Practices of Funding Organizations for Public Health Research and Practice: A Composite Narrative From Female, BIPOC Junior Researchers in Public Health
Source: Health Promot Pract. 2022 Oct 29;24(1):45–58. doi: 10.1177/15248399221129864 (PMC9806480; doi:10.1177/15248399221129864)

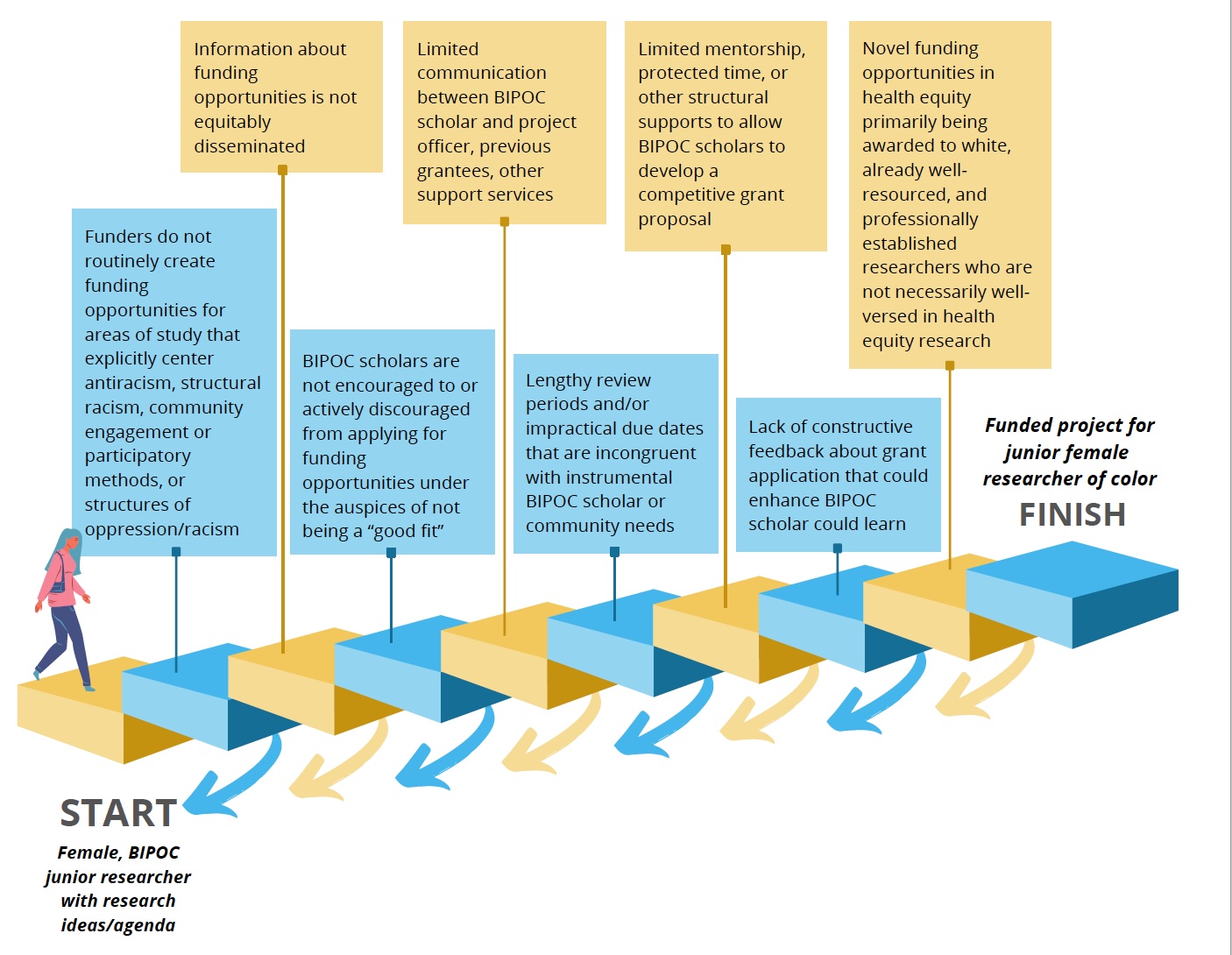

Supplement: sj-jpg-1-hpp-10.1177_15248399221129864 – Supplemental material for Examining the White Supremacist Practices of Funding Organizations for Public Health Research and Practice: A Composite Narrative From Female, BIPOC Junior Researchers in Public Health [file sj-jpg-1-hpp-10.1177_15248399221129864.jpg]

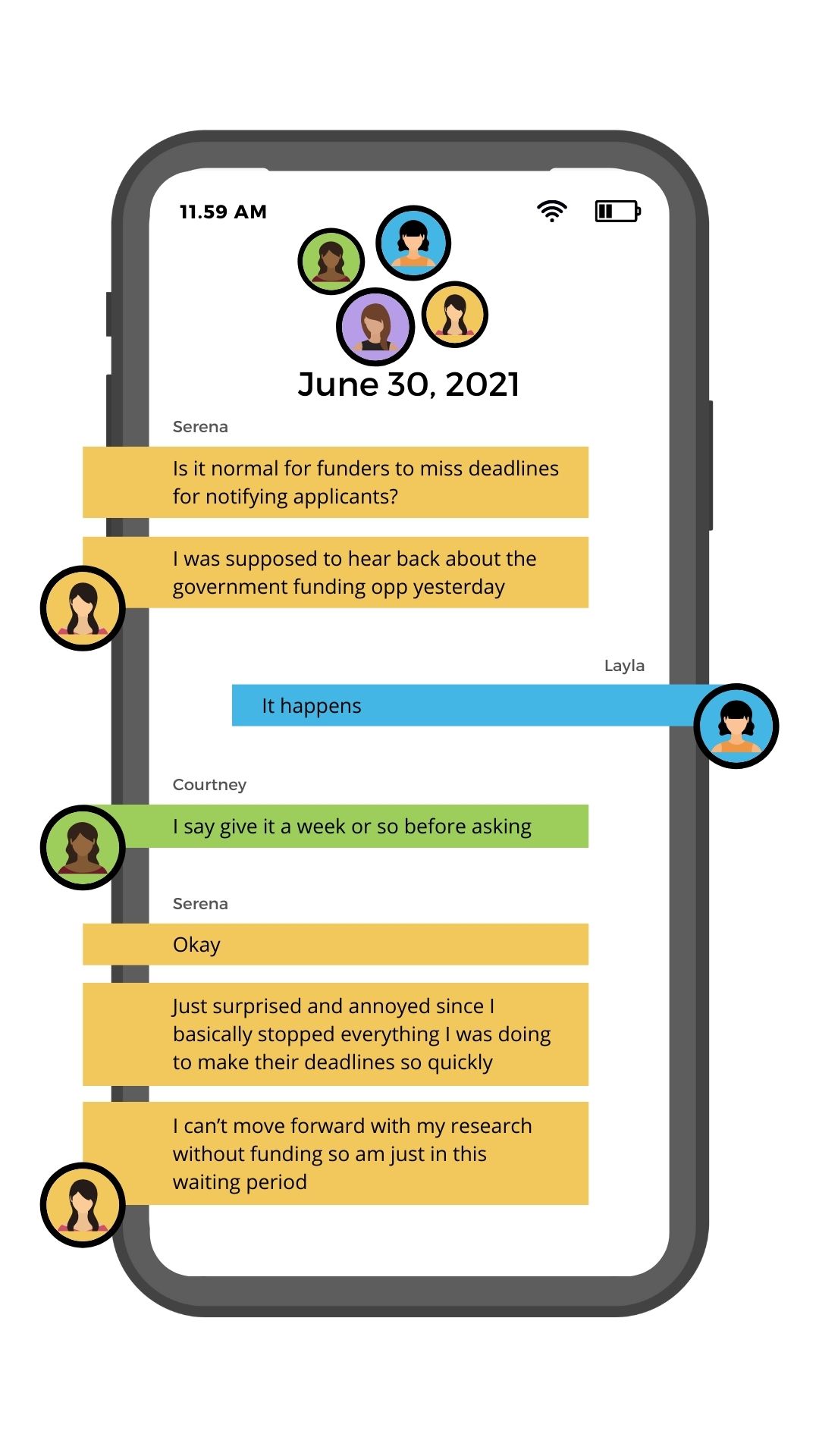

Supplement: sj-jpg-10-hpp-10.1177_15248399221129864 – Supplemental material for Examining the White Supremacist Practices of Funding Organizations for Public Health Research and Practice: A Composite Narrative From Female, BIPOC Junior Researchers in Public Health [file sj-jpg-10-hpp-10.1177_15248399221129864.jpg]

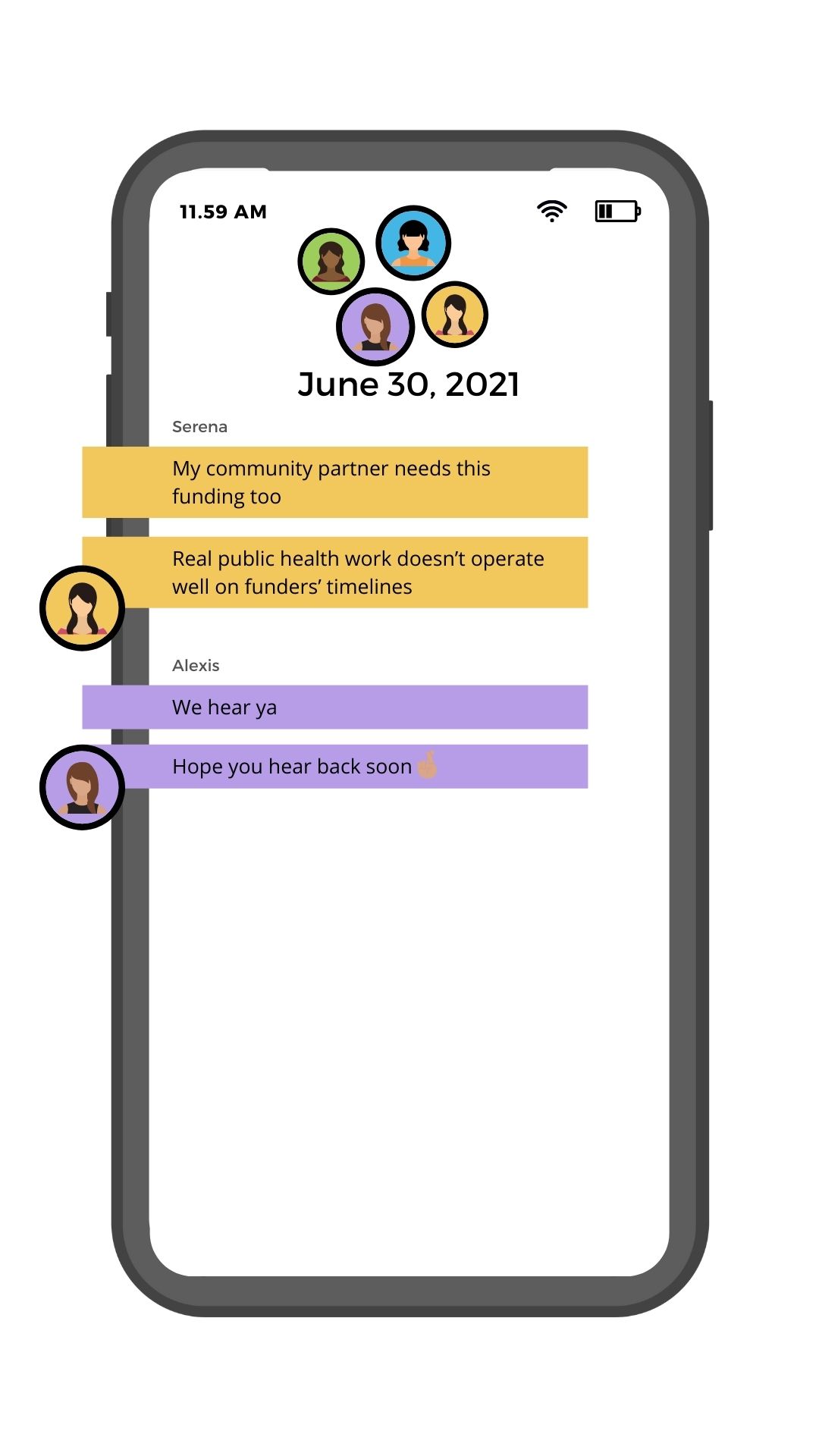

Supplement: sj-jpg-11-hpp-10.1177_15248399221129864 – Supplemental material for Examining the White Supremacist Practices of Funding Organizations for Public Health Research and Practice: A Composite Narrative From Female, BIPOC Junior Researchers in Public Health [file sj-jpg-11-hpp-10.1177_15248399221129864.jpg]

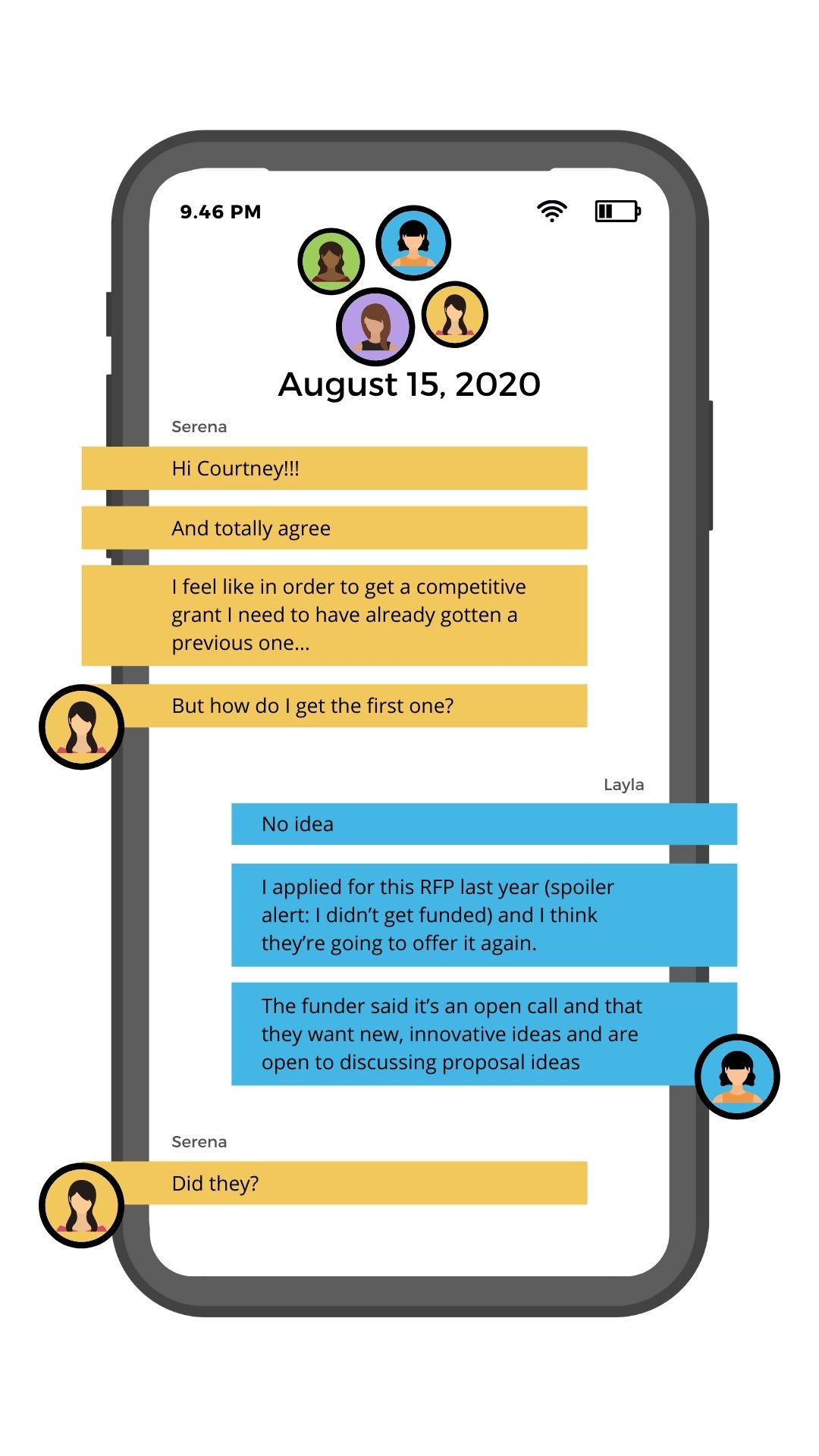

Supplement: sj-jpg-2-hpp-10.1177_15248399221129864 – Supplemental material for Examining the White Supremacist Practices of Funding Organizations for Public Health Research and Practice: A Composite Narrative From Female, BIPOC Junior Researchers in Public Health [file sj-jpg-2-hpp-10.1177_15248399221129864.jpg]

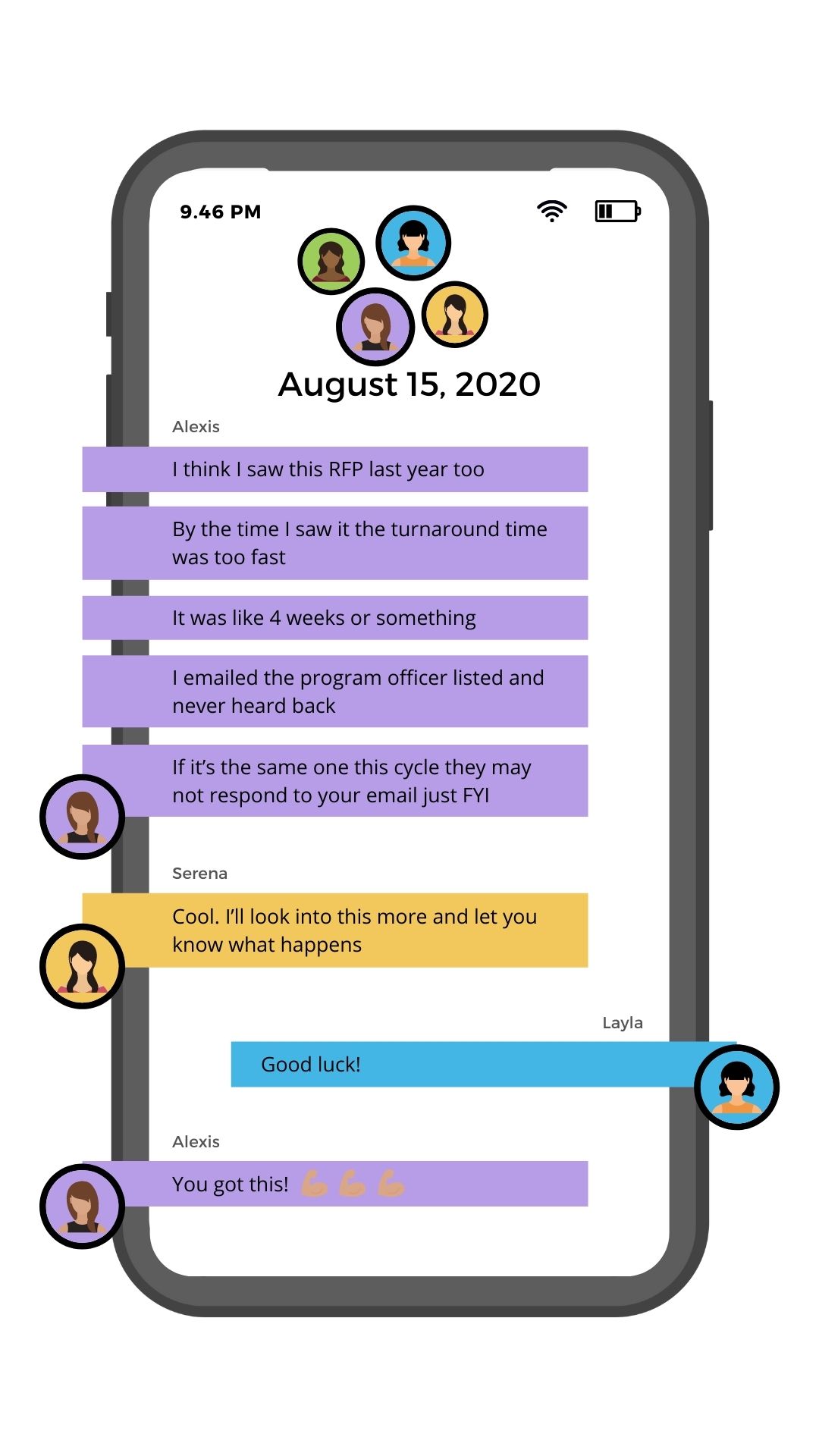

Supplement: sj-jpg-4-hpp-10.1177_15248399221129864 – Supplemental material for Examining the White Supremacist Practices of Funding Organizations for Public Health Research and Practice: A Composite Narrative From Female, BIPOC Junior Researchers in Public Health [file sj-jpg-4-hpp-10.1177_15248399221129864.jpg]

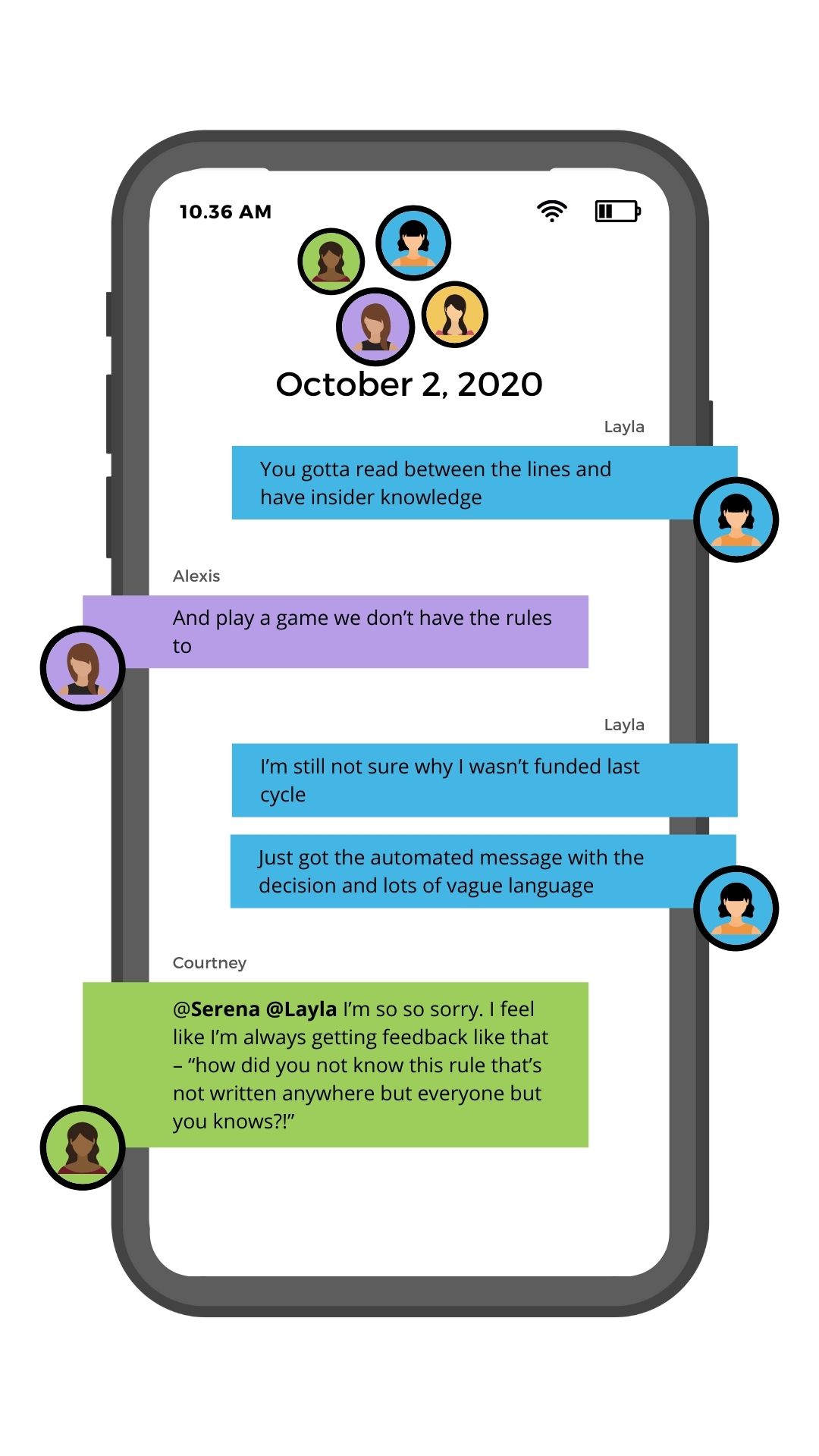

Supplement: sj-jpg-6-hpp-10.1177_15248399221129864 – Supplemental material for Examining the White Supremacist Practices of Funding Organizations for Public Health Research and Practice: A Composite Narrative From Female, BIPOC Junior Researchers in Public Health [file sj-jpg-6-hpp-10.1177_15248399221129864.jpg]

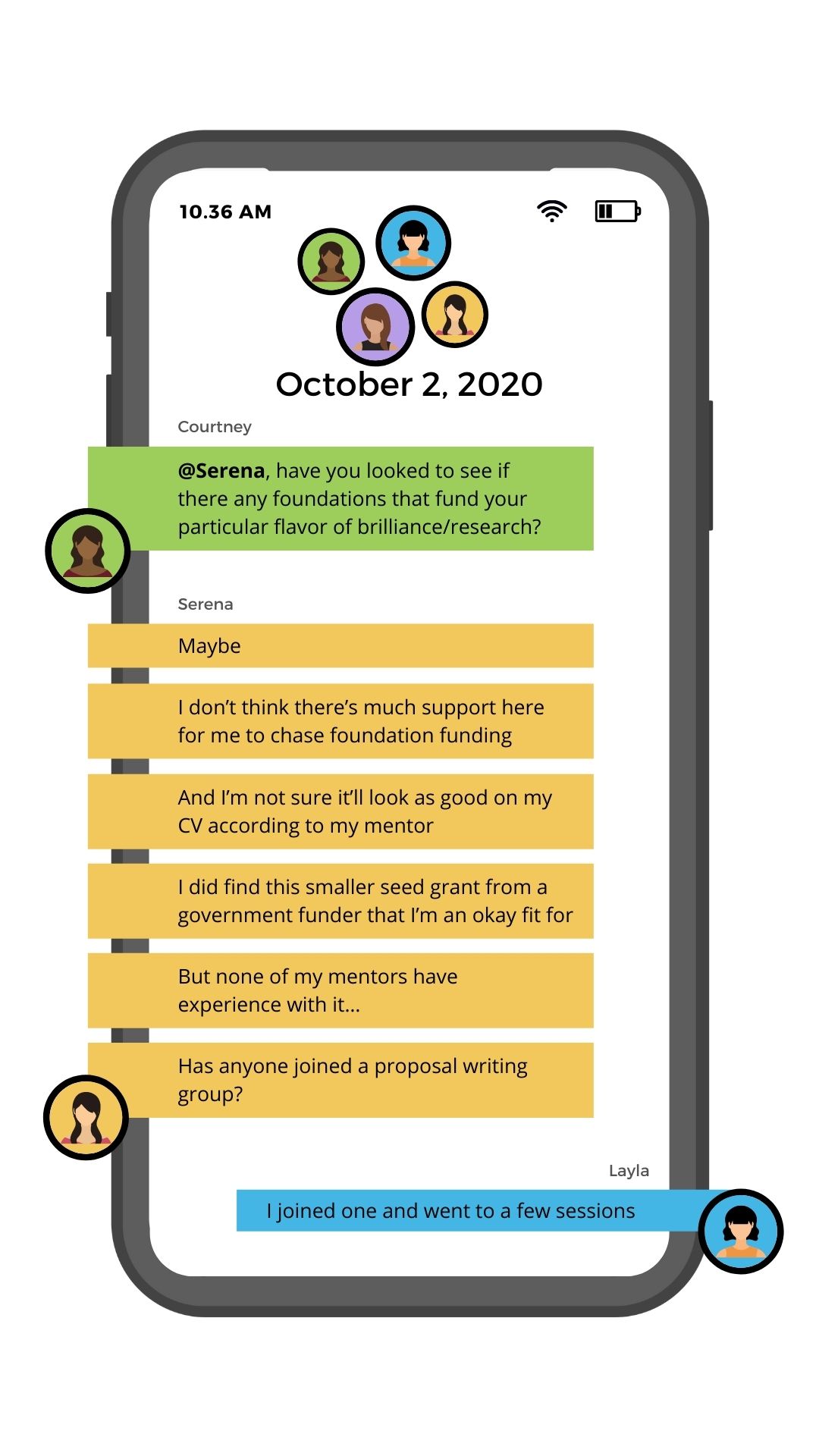

Supplement: sj-jpg-7-hpp-10.1177_15248399221129864 – Supplemental material for Examining the White Supremacist Practices of Funding Organizations for Public Health Research and Practice: A Composite Narrative From Female, BIPOC Junior Researchers in Public Health [file sj-jpg-7-hpp-10.1177_15248399221129864.jpg]

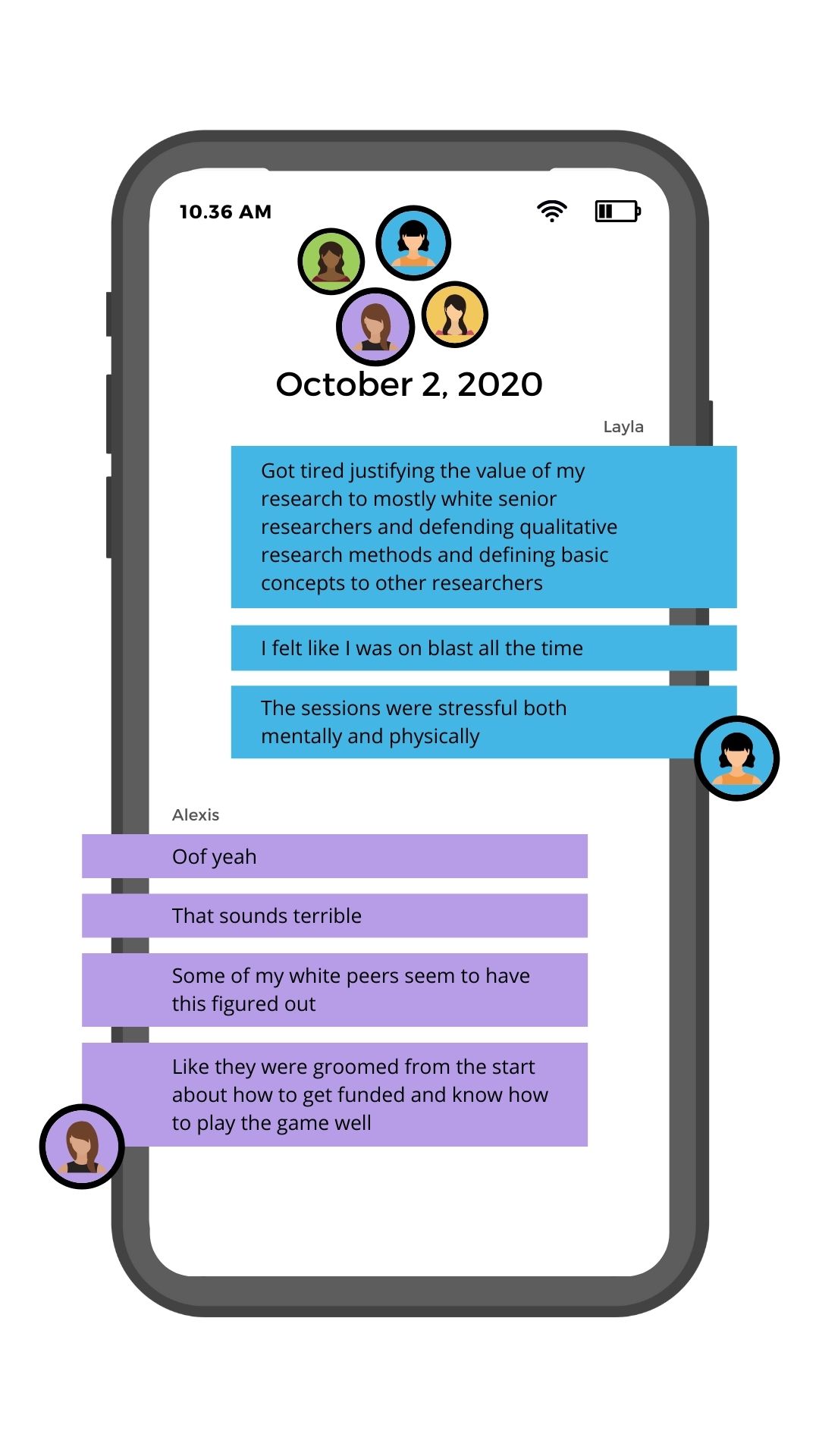

Supplement: sj-jpg-8-hpp-10.1177_15248399221129864 – Supplemental material for Examining the White Supremacist Practices of Funding Organizations for Public Health Research and Practice: A Composite Narrative From Female, BIPOC Junior Researchers in Public Health [file sj-jpg-8-hpp-10.1177_15248399221129864.jpg]

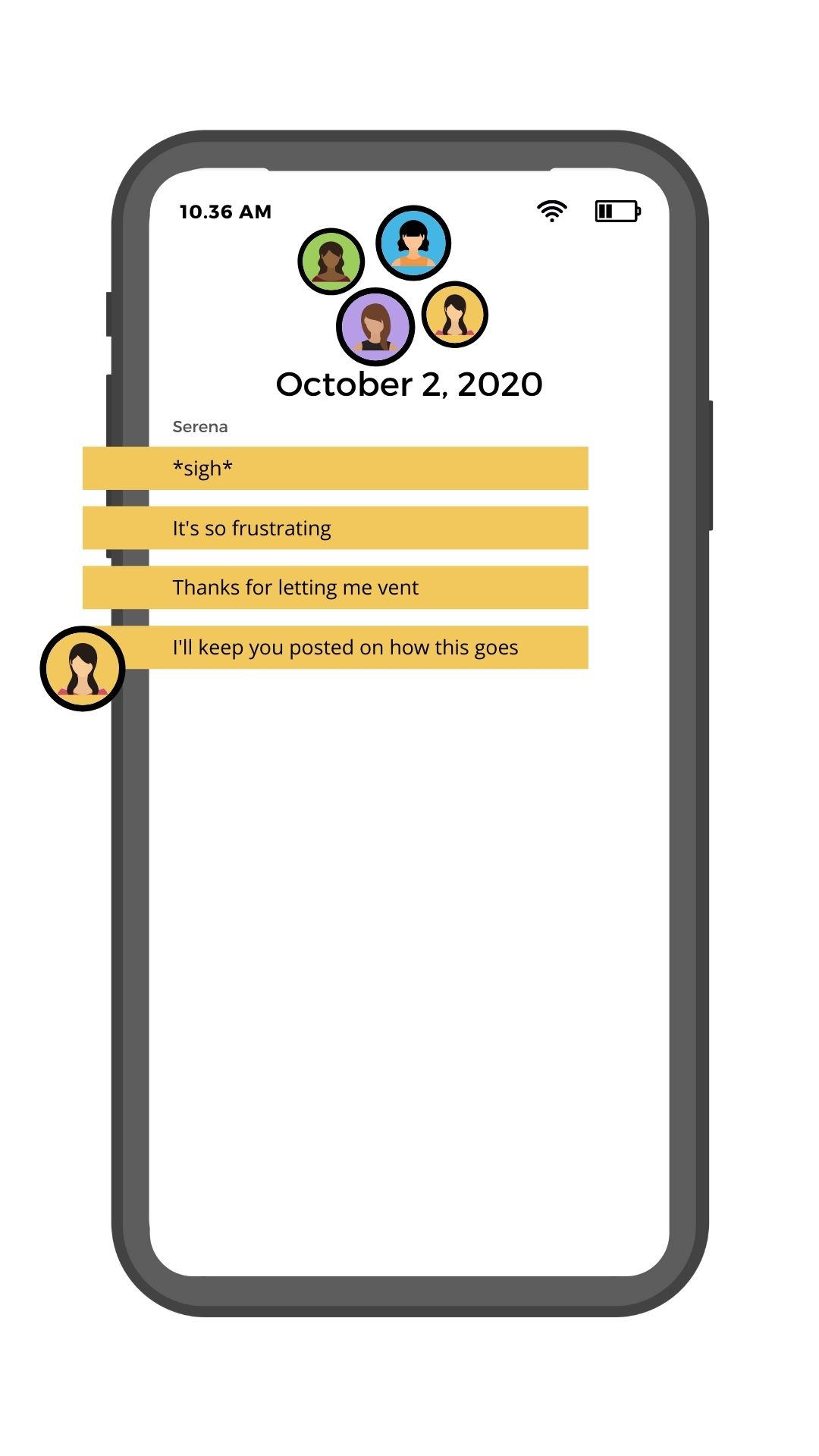

Supplement: sj-jpg-9-hpp-10.1177_15248399221129864 – Supplemental material for Examining the White Supremacist Practices of Funding Organizations for Public Health Research and Practice: A Composite Narrative From Female, BIPOC Junior Researchers in Public Health [file sj-jpg-9-hpp-10.1177_15248399221129864.jpg]
